# Ethnicity influences the gut microbiota of individuals sharing a geographical location: a cross-sectional study from a middle-income country

**DOI:** 10.1038/s41598-021-82311-3

**Published:** 2021-01-29

**Authors:** Jacky Dwiyanto, M. H. Hussain, D. Reidpath, K. S. Ong, A. Qasim, S. W. H. Lee, S. M. Lee, S. C. Foo, C. W. Chong, Sadequr Rahman

**Affiliations:** 1 School of Science, Monash University Malaysia, Jalan Lagoon Selatan, 47500 Bandar Sunway, Selangor Darul Ehsan Malaysia; 2 Health System and Population Studies Division, International Centre for Diarrhoeal Disease Research, Bangladesh, Dhaka, Bangladesh; 3 South East Asia Community Observatory, Segamat, Malaysia; 4 Genomics Facility, Monash University Malaysia, Bandar Sunway, Malaysia; 5 School of Pharmacy, Monash University Malaysia, Bandar Sunway, Malaysia; 6 Tropical Medicine and Biology Platform, Monash University Malaysia, Bandar Sunway, Malaysia

**Keywords:** Microbiology, Metagenomics, Microbiome

## Abstract

No studies have investigated the influence of ethnicity in a multi-ethnic middle-income country with a long-standing history of co-habitation. Stool samples from 214 Malaysian community members (46 Malay, 65 Chinese, 49 Indian, and 54 Jakun) were collected. The gut microbiota of the participants was investigated using 16S amplicon sequencing. Ethnicity exhibited the largest effect size across participants (PERMANOVA Pseudo-F = 4.24, R^2^ = 0.06, p = 0.001). Notably, the influence of ethnicity on the gut microbiota was retained even after controlling for all demographic, dietary factors and other covariates which were significantly associated with the gut microbiome (PERMANOVA Pseudo-F = 1.67, R^2^ = 0.02, p = 0.002). Our result suggested that lifestyle, dietary, and uncharacterized differences collectively drive the gut microbiota variation across ethnicity, making ethnicity a reliable proxy for both identified and unidentified lifestyle and dietary variation across ethnic groups from the same community.

## Introduction

A host of studies has established the differences in gut microbiota composition across different communities, which is attributable to a myriad of factors, from urban development^1^ to regional^2^ and seasonal variations^3,4^. However, a closer look has also revealed significant variations within members of the same community, which is attributed to differences in ethnicity. Deschasaux and colleagues^5^ observed differences between Caucasian and immigrant groups in the Netherlands, while Brooks and colleagues^6^ observed variations of the gut microbiota across the USA population. More recently, Peters and colleagues^7^ found that variations in the gut microbiome across ethnicity likely resulted from incomplete acculturation of migrant groups. Notably, all these studies have been conducted in a high-income country, where difference across ethnicity was partly attributable to recent migration events and their incomplete assimilation into the host nation. Probing further, Brooks and colleagues^6^ grouped all Asian into one large “Asian cluster” and the “South-Asian Surinamese” group described by Deschasaux and colleagues^5^ was a large group consisting of Indian and Javanese descents, while the Asian group described by Peters and colleagues mostly consisted of those with Korean ancestry (91%). Such pragmatic grouping ignored the fact that “Asians” consisted of genetically and culturally diverse communities.

Malaysia is a middle-income country that comprises four major ethnic groups: Malay, Chinese, Indian, and indigenous tribes of varying origins. Culturally, Malaysia represents a melting pot of multiple cultures and lifestyles introduced by its multi-ethnic population, forming a co-habiting cross-cultural community with parallel lifestyles^8,9^. This sets Malaysia as a valuable setting to investigate the impact of ethnicity across communities. Previously, studies on the effect of ethnicity on the GM of Malaysians have resulted in differing outcomes^10,11^. Chong and colleagues^10^ identified that ethnicity played a major role in influencing the GM of pre-adolescents from two ethnic groups in a closely located rural setting in Malaysia. In contrast, Khine and colleagues^11^ proposed that diet exerted a stronger influence on the GM than ethnicity alone in a comparison across Chinese children from Malaysia and southern China. To date, the effects of ethnicity on the GM of adult Malaysians have not been examined. In this paper, we present new insights into the impact of ethnicity on the adult GM in a multi-ethnic community from a single district in southern peninsular Malaysia.

## Methodology

### Study cohort

South East Asia Community Observatory (SEACO) is a health and demography surveillance system operating within Segamat, a district in the southern state of Johor, Malaysia^12^. The economy of Segamat is mainly supported by private agriculture. The ethnicity distribution of the cohort under SEACO is comparable to the national average, but with a slightly higher proportion for both the young (< 20 years old) and elderly (> 65 years old) due to the outflow of young adults for better job and education opportunities. This study was conducted on a subset of community participants under SEACO’s surveillance. Human ethics approval was obtained from the Monash University Human Research Ethics Committee (MUHREC, project no: 1516), which is per the Declaration of Helsinki, and complied with international and institutional standards. All participants provided written informed consent and were compensated with a lunchbox and umbrella as tokens of appreciation. Written informed consent was also obtained from the parent of the participants who were under 18 years old during the sampling period.

### Community participants

Participants of four major ethnic groups were recruited in equal proportion from three sub-districts in Segamat. Each ethnicity predominantly resided in individual sub-districts in Segamat except for Chinese and Jakun, both of whom lived in the Bekok sub-district. Jakun, however, resided in a more rural setting, across the fringes of the jungle, while the other ethnicities lived in the semi-urbanized part of town. A total of 458 participants residing in 144 households were recruited from May through June 2018.

### Collection of lifestyle and dietary habit

Participants were interviewed by trained SEACO data collectors to obtain information on their dietary, lifestyle, and medical history. Participants below the age of 17 provided their answers under parental guidance. Besides that, the height, weight, and blood pressure of the participants were also measured.

### Gut microbiota analysis

Each participant was provided with a Fisherbrand Commode Specimen Collection System and a cooler box containing cooler packs to store their fecal samples. The fecal consistency was self-measured by participants assisted with a provided Bristol Stool Scale (BSS) chart. The fecal samples were collected and transported back to the laboratory the next day, where they were kept at − 50 °C until further analysis.

DNA extraction was conducted on the collected fecal samples from April through June 2019 using protocol Q of the International Human Microbiome Standards^13^. Briefly, fecal DNA extraction was conducted using the QIAamp DNA Stool Mini Kit in combination with a zirconium bead-beating method. The V3-V4 region of the 16S rRNA gene was then amplified using the primer pair 341F and 805R. The metagenomic library was then prepared according to the Illumina 16S Metagenomic Sequencing Library Preparation and amplified using the Illumina Miseq platform with 2 × 250 bp paired-end sequencing.

The raw sequence data yielded 16,723,707 reads, with a mean of 72,397 reads per sample. Primers and barcode sequences were then removed using the R package DADA2^14^, yielding 14,548,208 reads, with a mean of 62,979 reads per sample. After inferring amplicon sequence variants using DADA2, the abundance of each sequence variant was exported into Pathway Prediction by Phylogenetic Placement (PAPRICA) pipeline version 0.5^15^ for phylogenetic placement. In brief, the sequences were aligned and placed into the near-full-length 16S rRNA gene reference tree. Phylogenetic groupings, or “edges,” was inferred based on the placement of the sequences in the reference tree. An edge can be referring to either the terminal node of the tree, or a path connecting two nodes. Unique numbers were assigned to each edge, and the number of consensus sequences in the same edge was recorded into the abundance table for further downstream analyses. Similarly, the abundance data of each edge was utilized for the prediction of metabolic pathway abundance by feeding them into the reference MetaCyc metabolic pathway database^16^.

### Statistical analyses

Count data were filtered to exclude edges and pathways with less than 1000 counts prior to the analyses. The α-diversity indices of Shannon diversity and Pielou’s evenness were inferred using the phyloseq package version 1.30.0^17^. For ß-diversity analysis, the edge and pathway data containing the bacterial taxa abundance profiles was transformed using centered-log transformation using the function aldex.clr in the ALDEx2 R package version 1.22.0^18^, to account for the compositionality of high throughput sequencing data^19^. The transformed data was then ordinated using principal component analysis with Euclidean distance using the R package vegan version 2.5-6^20^. Afterward, the differences across factors were analyzed using Permutational Multivariate Analysis of Variance (PERMANOVA) implemented in the adonis function under the R package vegan version 2.5-6^20^. The *Prevotella*: *Bacteroides* ratio (P:B) and its 95% confidence interval was analyzed using linear mixed model by inputting the log-transformed P:B as the dependent variable with household as the random effect, to account for the household clustering of the participants, using the package lme4 version 1.1-21^21^. The presence of gut enterotypes were analyzed using the R package DirichletMultinomial version 1.28.0^22^. The differential abundance of taxa across factors was analyzed using the generalized linear model wrapped in the R package ALDEx2 version 1.22.0^18^, with p-value for multiple-groups comparison adjusted using the Benjamini–Hochberg method with a threshold for significance of 0.1.

## Results

### Population demographics

This study recruited participants from Segamat, a district located in southern Peninsular Malaysia. Fecal samples from 231 participants in 110 families were obtained. After excluding participants with missing data, 214 participants from 106 families were included for analyses. The participants were equally distributed across sex and ethnicity (Table 1). There was an equal occupational distribution across ethnicity, with most participants (n = 60) being homemakers. Every ethnic group also exhibited similar economic status, with most households in each ethnic group earning between RM1001–5000 monthly. However, a significantly higher proportion of Jakun and Chinese earned the least (RM401–700) and the most (> RM5000), respectively. Overall, the mean age of the participants was 44.25 ± 19.59, ranging from 10 to 83 years old. A higher proportion of Chinese and Jakun belonged to the older and younger age quartiles, respectively (Chi-Square test, p < 0.05, Supplementary Table S1). Meanwhile, there was an equal number of Indian and Malay participants across the age groups (Chi-Square test p > 0.05).

**Table 1:** Demographic distribution of community participants in Segamat, southern Malaysia.

Factors	PERMANOVA		Level	Chinese		Jakun		Indian		Malay		N	Chi-sq
	R^2^	p-value		n	%	n	%	n	%	n	%		
Ethnicity	0.06	0.00	n/a	65	0.3	54	0.25	49	0.23	46	0.21	214	0.27
Sex	0.00	0.67	M	28	0.29	21	0.22	24	0.25	23	0.24	96	0.78
			F	37	0.31	33	0.28	25	0.21	23	0.19	118	0.22
Age	0.02	0.01	10–28	12	0.22	22	0.41	7	0.13	13	0.24	54	0.03
			29–48	7	0.13	20	0.36	18	0.33	10	0.18	55	0.04
			49–59	23	0.45	6	0.12	12	0.24	10	0.19	51	0.01
			60–83	23	0.43	6	0.11	12	0.22	13	0.24	54	0.01
Subdistrict	0.03	0.00	Bekok	65	0.55	54	0.45	0	0	0	0	119	0.00
			Chaah	0	0	0	0	49	1	0	0	49	0.00
			Jabi	0	0	0	0	0	0	46	1	46	0.00
Income (MYR)	0.02	0.07	< 400	1	0.33	2	0.67	0	0	0	0	3	0.3
			401 – 700	4	0.16	12	0.48	7	0.28	2	0.08	25	0.03
			701 – 1000	12	0.57	3	0.14	6	0.29	0	0	21	0.00
			1001 – 5000	41	0.27	36	0.23	33	0.21	44	0.29	154	0.59
			> 5000	7	0.64	1	0.09	3	0.27	0	0	11	0.02
Occupation	0.06	0.17	Agricultural	18	0.42	12	0.28	4	0.09	9	0.21	43	0.02
			Children	10	0.37	5	0.19	2	0.07	10	0.37	27	0.07
			Craft	0	0.00	1	0.25	2	0.50	1	0.25	4	0.57
			Elementary	0	0.00	1	0.20	2	0.40	2	0.40	5	0.53
			Homemaker	14	0.23	18	0.30	15	0.25	13	0.22	60	0.82
			Operator	1	0.33	0	0.00	2	0.67	0	0.00	3	0.30
			Others	0	0.00	0	0.00	3	1.00	0	0.00	3	0.03
			Professional	1	0.50	0	0.00	1	0.50	0	0.00	2	0.57
			Self-employed	3	0.33	2	0.22	1	0.11	3	0.33	9	0.75
			Service	5	0.28	0	0.00	8	0.44	5	0.28	18	0.06
			Technician	0	0.00	1	1.00	0	0.00	0	0.00	1	0.39
			Unemployed	12	0.32	14	0.37	9	0.24	3	0.08	38	0.06

### α-Diversity was associated with BSS, BMI, and income, but not ethnicity

**Figure 1:**
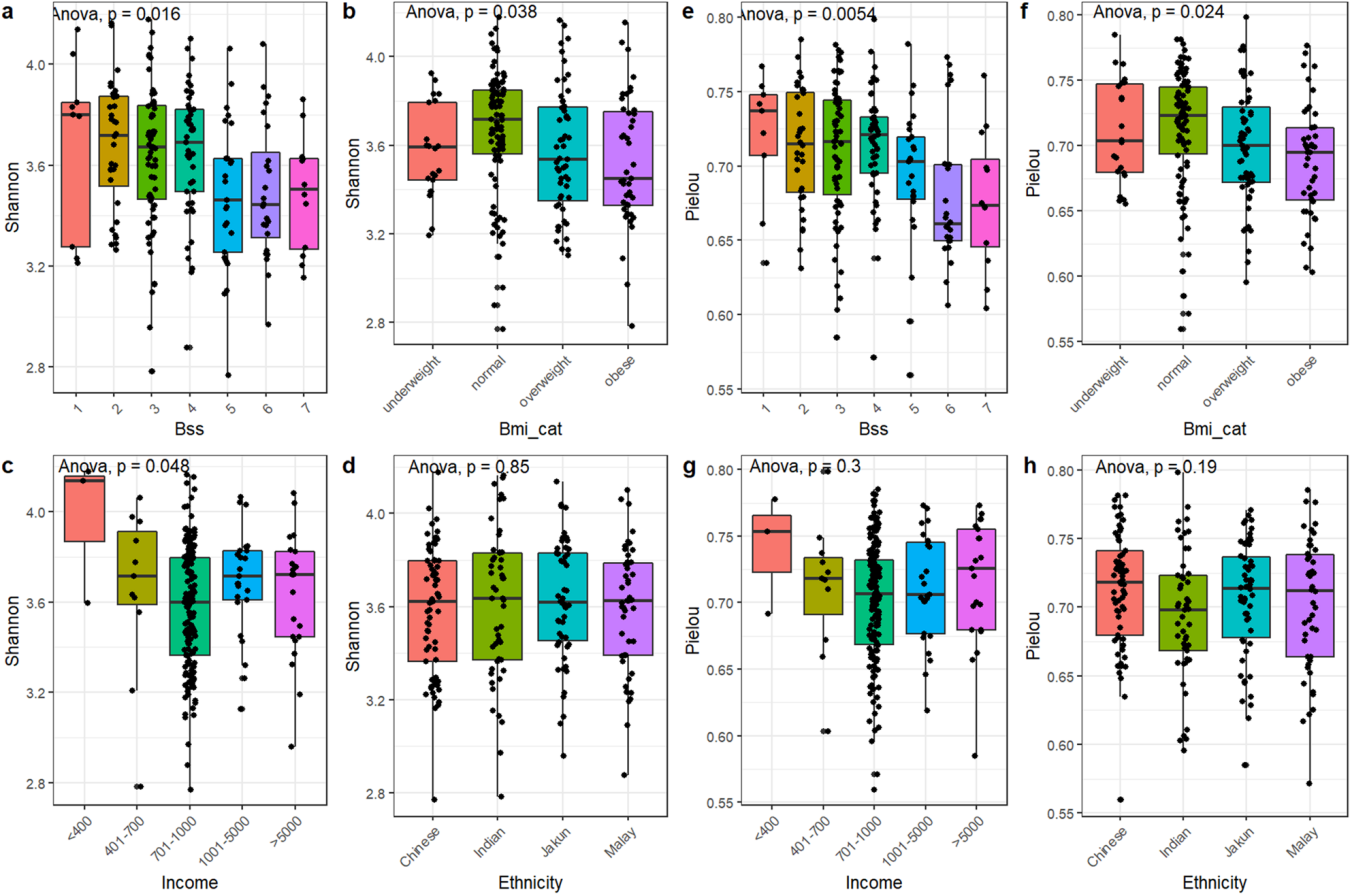
Shannon (**a**) and Pielou’s (**b**) α-diversity indices across Bristol stool scale, body mass index, household income, and ethnicity with ANOVA statistical comparison.

A total of 39 lifestyle factors were recorded through the administered survey (Supplementary Table S2). These factors were tested against the Shannon diversity index, which measures the richness and proportion of different bacterial taxa, and Pielou’s index, which measures the evenness in the distribution of the taxa abundances. Bristol stool scale (BSS) (Fig. 1a,e) and BMI (Fig. 1b,f) exhibited significant differences across both indices, showing a negative association with diversity (BSS scale 3 vs. scale 6; BMI healthy vs. obese, Tukey’s HSD test, p < 0.05). Household income was significantly associated with diversity but had similar evenness (Fig. 1c,g). Participants in the lower-income group exhibited a higher diversity, although Tukey’s HSD test found no significance after correcting for multiple testing. Meanwhile, diversity and evenness were similar across ethnicity (Fig. 1d,h, p > 0.05).

### Ethnicity exhibited the strongest effect on the gut microbiota

**Figure 2:**
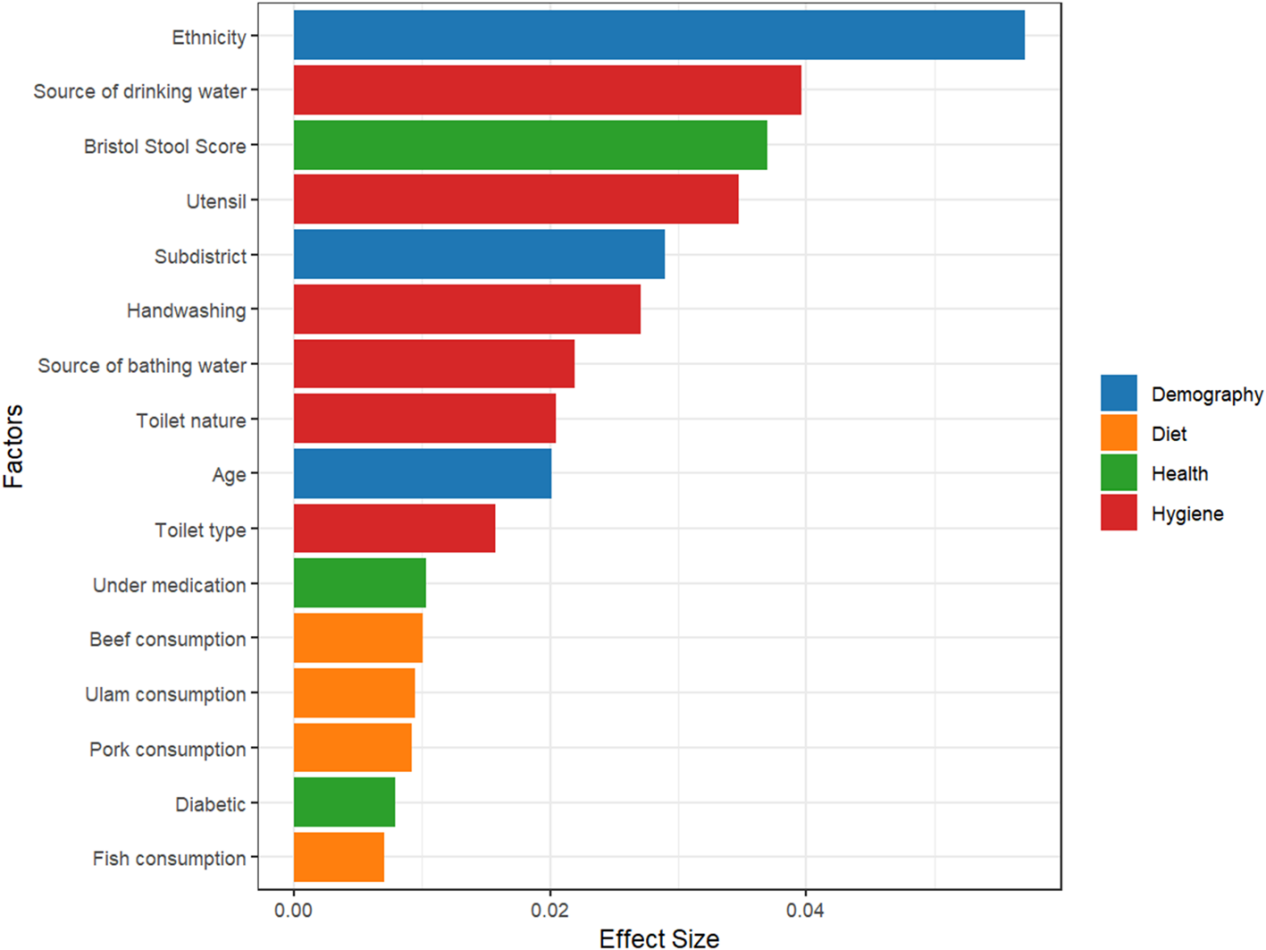
Factors significantly associated (p < 0.05) with the gut microbiome and their effect sizes determined through univariate PERMANOVA analysis.

**Figure 3:**
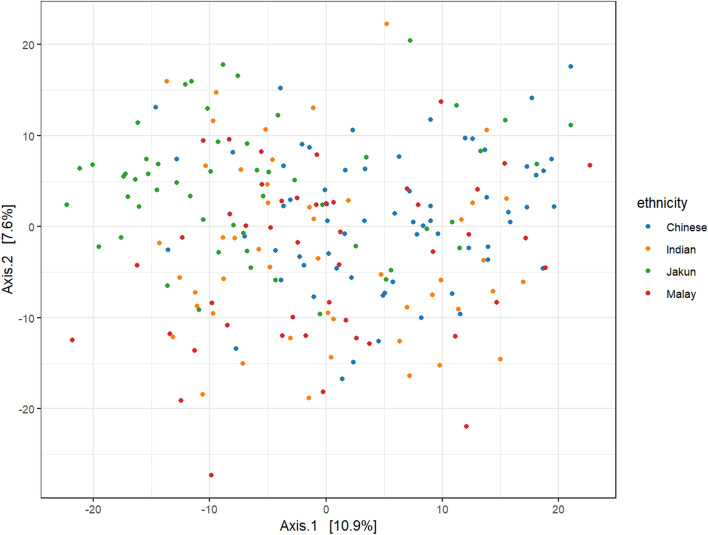
Principal coordinate analysis of the Segamat participants classified based on their ethnicity, ordinated using Euclidean distance matrix.

PERMANOVA univariate analysis identified 16 factors that were significantly associated with the GM (p < 0.05). These factors can be categorized into either demographic, dietary behavior, hygiene practices, or health conditions (Supplementary Table S2, Fig. 2). Altogether, these factors accounted for 23.49% of the variation observed in the GM. Among the factors analysed by univariate analyses, ethnicity exerted the largest effect size (Fig. 3, PERMANOVA Pseudo-F = 4.24, R^2^ = 0.06, p = 0.001). A multivariate analysis was then conducted, which revealed that ethnicity was still a significant influencer of the GM albeit at a smaller effect size after adjusting for demographic covariates (age, BMI, sex, income, occupation) (PERMANOVA Pseudo-F = 3.27, R^2^ = 0.05, p = 0.001). Inclusion of additional identified cofactors reduced the effect size further, but ethnicity was still significant (PERMANOVA Pseudo-F = 1.67, R^2^ = 0.02, p = 0.002). Additionally, the significance of ethnicity was retained when the analysis was redone on a subset of participants controlled for either household earning between RM1000–5000 (n = 154, Pseudo-F = 4.09, R^2^ = 0.08, p = 0.001), having indoor piped water source (n = 187, Pseudo-F = 3.72, R^2^ = 0.05, p = 0.001), red meat consumption (n = 136, Pseudo-F = 2.84, R^2^ = 0.06, p = 0.001), or health (n = 47, Pseudo-F = 1.53, R^2^ = 0.10, p = 0.015). The healthy group consisted of equal representation of participants from each ethnic group who had healthy BP, BMI, and BSS, along with the absence of any history of chronic disease and medication use (Supplementary Table S3).

### Ethnicity-specific microbiota and their association with host demographics, lifestyle, diet, and hygiene

**Figure 4:**
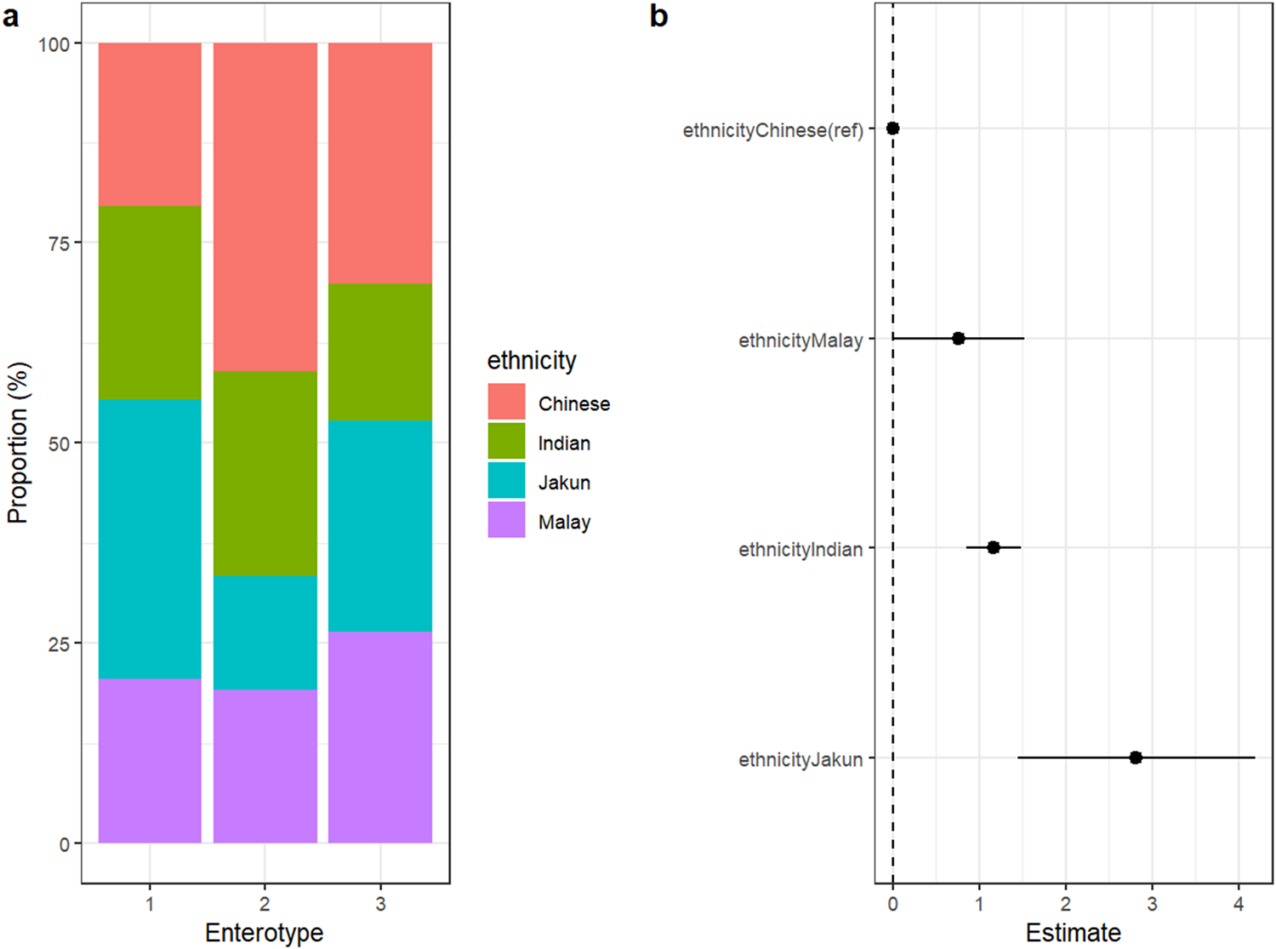
Multi-ethnic comparison of (**a**) gut enterotype profiles analyzed using Dirichlet Multinomial Model, and (**b**) *Prevotella:Bacteroides* ratio analyzed using linear mixed model (likelihood ratio test p < 0.05).

In a previous study, Arumugam and colleagues^23^ suggested that the human microbiome can be broadly stratified into three enterotypes: *Bacteroides*, *Prevotella*, and *Ruminococcus*-dominant. Using Dirichlet Multinomial model^22^, three clusters were detected in our dataset, namely *Prevotella*-dominant, *Bacteroides*-dominant, and *Bifidobacterium*-dominant enterotypes (Fig. 4a, Supplementary Fig. S1). Interestingly, a higher proportion of the *Prevotella-* (type 1) and *Bacteroides-* (type 2) dominant enterotypes were exhibited by Jakun and Chinese, respectively. Both Malay and Indian were equally distributed in the first and second enterotype. Supporting this observation, a linear mixed model analysis on the ratio of *Prevotella* to *Bacteroides* (P:B) resulted in a gradient, with Jakun, Indian, Malay and Chinese exhibiting the largest to the lowest P:B ratio (Fig. 4b) (likelihood ratio test = 0.002).

**Figure 5:**
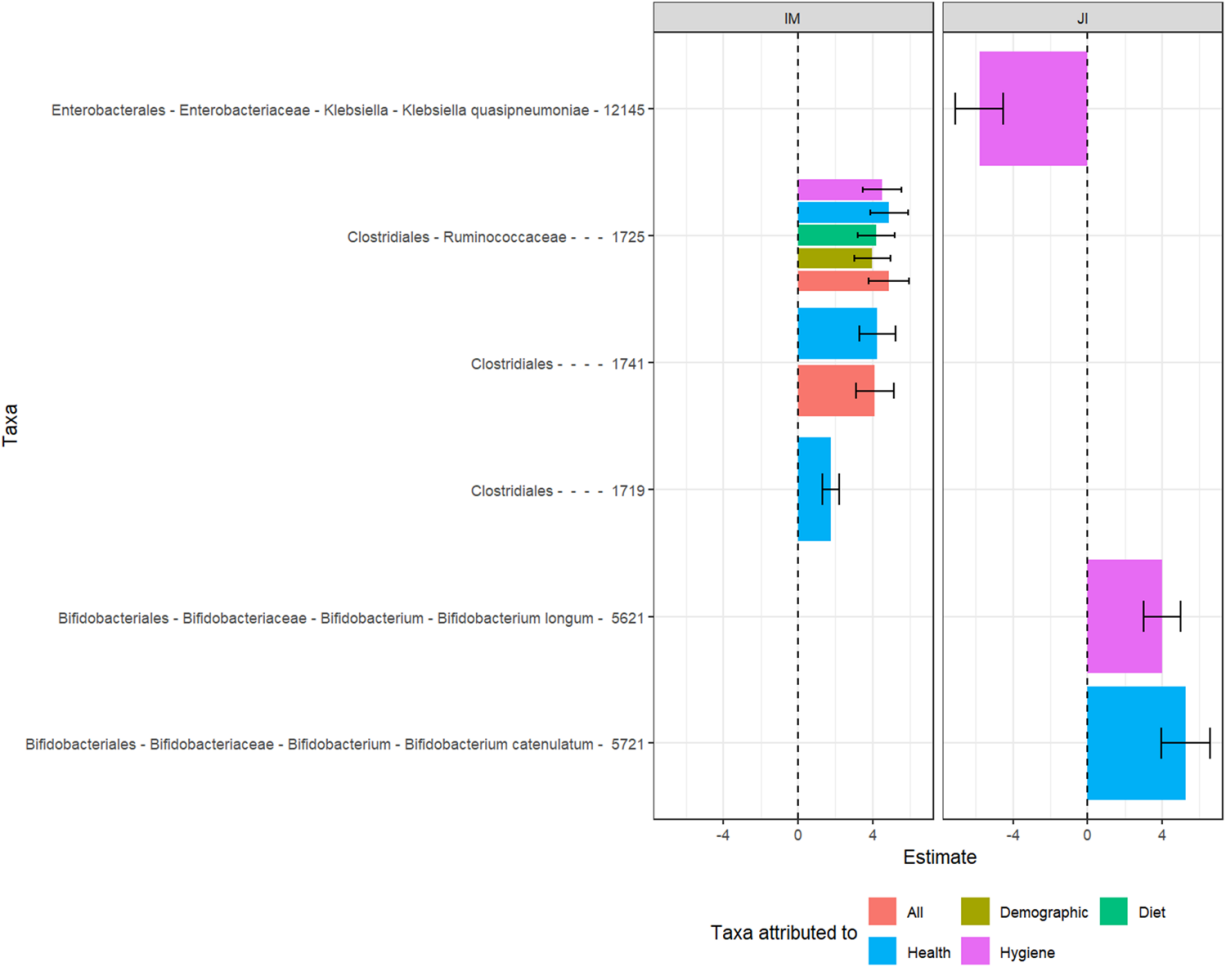
Taxa which were significantly differentially abundant across ethnicity in Segamat as analyzed using ALDEx2 generalized linear model (FDR < 0.1). Description: *IM* Indian–Malay, *JI* Jakun–Indian.

To identify taxa which were associated with specific covariate indices (either demographic, dietary, health or hygiene), we conducted differential abundance analyses which were controlled for all significant covariates detected by the univariate PERMANOVA analysis (Fig. 2) except the index of interest (Fig. 5). Additionally, the demographic and dietary covariates' adjustment included other variables such as occupation and fruits intake (Supplementary Table S2) to account for dietary and demographic factors being potential major confounders to the gut microbiota^24,25^. Significant differences were identified for the Indian-Malay and Jakun-Indian ethnic pairings, where Malay exhibited an elevated abundance of two unclassified Clostridiales (edge 1741 FDR = 0.01; edge 1719, FDR = 0.06) associated with the hygiene index. Meanwhile, Jakun and Indian were differentiated by hygiene index, with the former associated with a higher prevalence of *Klebsiella quasipneumoniae* (edge 12,145, FDR = 0.03) in the former and *Bifidobacterium longum* (edge 5621, FDR = 0.05) in the latter. Additionally, *Bifidobacterium catenulatum* (edge 5721, FDR = 0.09) was also elevated in Indian, which was associated with health covariates. Interestingly, two edges of the Clostridiales order were found to be elevated in Malay compared to Indian even after adjusting for all these covariates (edge 1725, FDR = 0.03; edge 1741, FDR = 0.05).

**Figure 6:**
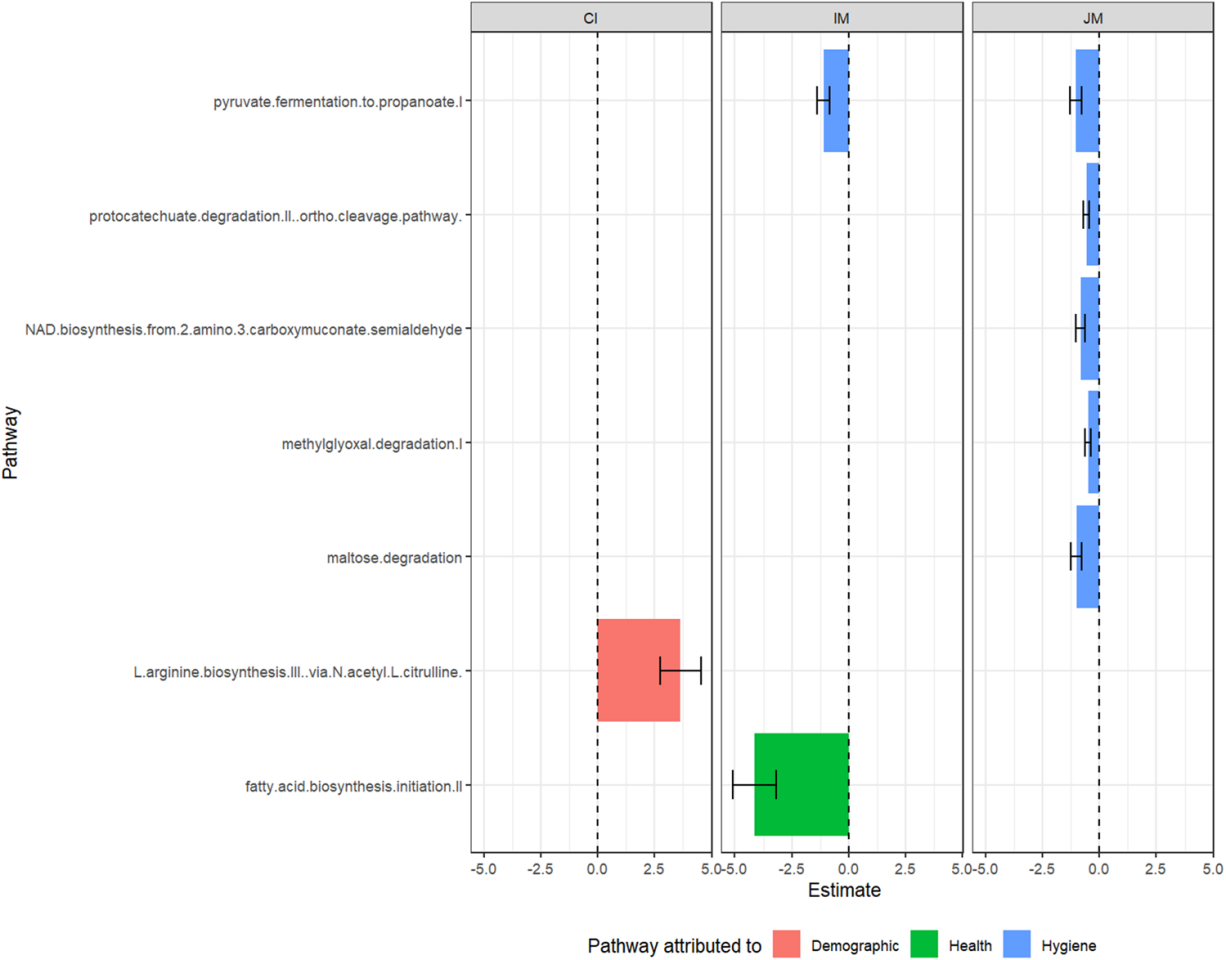
Predicted pathways which were significantly differentially abundant across ethnicity in Segamat as analyzed using ALDEx2 generalized linear model (FDR < 0.1). Description: *CI* Chinese–Indian, *IM* Indian–Malay, *JM* Jakun–Malay.

The functional metabolic pathways of the microbiota were also compared (Fig. 6). Notably, functional pathway differences between Jakun and Malay was attributed to hygiene covariates, with Jakun exhibiting elevated pathways relating to pyruvate fermentation, protocatechuate degradation, NAD biosynthesis, methylglyoxal degradation, and maltose degradation pathways. Meanwhile, Indians had a higher elevation of L-arginine and fatty acid biosynthesis pathways compared to Chinese and Malay, which was attributed to demographic and health index, respectively.

Substantial variations across lifestyle parameters were observed across the four ethnic groups (Supplementary Table S2). Chinese consumed the most pork, while Jakun and Malay had higher ulam (a group of traditional vegetables commonly eaten as delicacies or side dish) consumption compared to Chinese and Indian. Across hygiene indices, Jakun had the least access to piped water for both drinking and bathing. Also, a higher proportion of Jakun utilized borehole toilets compared to the other ethnic groups, who overwhelmingly used flush toilets. Additionally, Indian exhibited the highest diabetes burden in the dataset.

## Discussion

This study provides novel insights into the effect of ethnicity on the GM of a multi-ethnic community. In the absence of geographical variation, ethnicity exerted a significant influence on the GM of participants from Segamat. Notably, the significance of ethnicity was retained after adjusting for demographic, dietary and other significant covariates, albeit at a smaller effect size.

It is worth disclosing the natural clustering of each ethnic group in different sub-districts, resulting in a collinear relationship between ethnicity and sub-district in our dataset. Jabi had the heaviest density of Malay and was located approximately 50 km away from both Bekok and Chaah, which were neighboring sub-districts. Nevertheless, all sub-districts were under the same Segamat municipality and had similar environmental exposure and demographic distribution^12^. As such, all statistical adjustments conducted in this study excluded the sub-district covariates despite its significance.

The significance of ethnicity on the GM was likely driven by the unique lifestyle patterns exhibited by each ethnic group as captured through the administered survey. Notably, the higher proportion of *Prevotella*-dominant enterotypes in Jakun was likely driven by higher consumption frequency plant-based foods as reflected in the higher consumption of *ulam* in Jakun. *Ulam* itself is a traditional salad which comprises multiple types of traditional vegetables and is widely consumed by the local community either as a delicacy or a side dish^26,27^. Importantly, *ulam* has been reported to contain high antioxidant properties^26^.

Additionally, differential abundance analyses indicated that the gut-ethnic variation observed could be attributed to either health or hygiene indices. In our dataset, three health indices were significantly associated with the GM: bristol stool scale, diabetes, and medication. Clostridiales has been previously inversely correlated with type II diabetes^28^, which likely drove their reduced abundance in Indian, considering that Indians had a significantly higher diabetes burden, both nationally^29^ and in our dataset. Meanwhile, the genus *Bifidobacterium* is frequently associated with the consumption of probiotics and fermented food^30^. It is therefore intriguing that two edges belonging to this genus were elevated in Indian due to health and hygiene indices, but not diet. As noted earlier, this could be due to the dietary variations which were not captured in this study. It is worth noting that *Bifidobacterium* is commonly reported to be elevated in South Asians, which includes Indians, both in infants and adults^5,31,32^. Regardless of whether this association is attributed to dietary or genetics, as hypothesized by others^5,32^, it is interesting to investigate the higher burden of diabetes in South Asian despite being consistently elevated in *Bifidobacterium*, which has been strongly associated with an alleviation of diabetes conditions^33^. Altogether, these outcomes suggested that “ethnicity” is a manifestation of multiple lifestyle factors, which might have a low effect singly, but collectively resulted in a unique GM profile that distinguished the ethnic groups in Segamat.

Interestingly, the influence of ethnicity was retained even after adjusting for all significant covariates, with differential abundance analysis observing the elevation of two Clostridiales edge in Malay compared to Indian (edge 1725, FDR = 0.03; edge 1741, FDR = 0.05). Previously, the influence of genetics on the GM has been hypothesized to be in the 1.9–8.1% range^34^. It remains to be seen whether part of this ethnic influence in our study was driven by genetic factors or they were merely a manifestation of lifestyle variables which were unaccounted for by this study.

Understanding predicted pathways could provide a better picture due to the functional roles of the GM^15,35^. Notably, the elevation of NAD biosynthesis pathway in Jakun could be related to their higher frequency of plant-based food consumption, where NAD biosynthesis has been associated with the presence of tryptophan^36^, a plant-derived amino acid^37^. The elevation of protocatechuate degradation could have arisen from the abundance of aromatic hydrocarbons in the Jakun’s gut environment^38^. Aromatic hydrocarbon is commonly found in leafy vegetables^39^. In addition, elevations of pyruvate and maltose metabolism suggested the higher carbohydrate consumption among Jakun. Meanwhile, the elevation of fatty acid biosynthesis in Indian suggests the abundance of precursor metabolites for fatty acid synthesis in their gut environment, which might be indicative of a high-fat dietary preference. This outcome could also be associated with the significantly higher BMI exhibited by the Indians in Segamat. It is worth noting that most of these functions were more closely associated with energy metabolism. It is possible that the non-exhaustive nature of our dietary survey likely resulted in the collinearity of these factors with non-dietary indices.

Urbanization has been associated with variations across α-diversity measures^1,2,40^. Higher intake of resistant starch and a plant-based diet is typical among rural populations compared to their urbanized counterpart, which typically had a diet high in protein and fat content. Even though Jakun resided in a more rural setting, in the fringes of the jungle, the similar economic status and availability of common foods precluded any major impact of urbanization in our samples. Davenport and colleagues^3^ had observed the lack of difference in the Shannon index among the self-isolating Hutterites across different seasons, which influenced the consumption of fresh fruit and vegetable produce during the summer and winter seasons. The absence of such a difference indicates the presence of other confounding factors exerting effects on bacterial diversity, which we have yet to establish.

The analysis of a subset of healthy participants exhibited the largest effect size of ethnicity. Among those excluded were diabetic, hypertensive, and obese participants, as these factors have been associated with changes in the GM^41–43^. Apart from diabetes, there was an equal proportion of hypertensive and obese participants across ethnicity. Thus, it was likely that their inclusion in the overall dataset partially masked the ethnicity effect. Participants suffering from the same conditions were expected to exhibit a similar GM profile, consequently clouding the variations across ethnicity. Our result of ethnicity-specific healthy GM is consistent with previous studies^35,44^. The mapping of core “healthy GM” is essential to pave the way for personalized medicine, given the strong implication of GM in the etiology and prognosis of various diseases.

A comparison of the gut microbiome between Chinese and Indian students have previously been conducted in Singapore^45^. *Prevotella* and *Lactobacillus* were more abundant among Indians, which was attributed to higher consumption of whole wheat by Indian students, along with the dominance of animal fat and protein in the diet of the Chinese students. Like our study, they found that Indian students exhibited a higher *Prevotella* abundance compared to Chinese. It is unclear whether the Chinese and Indian students in their study were of national or foreign origin. A difference in their nation of origin would be expected to introduce variation in their gut microbiome. More crucially, the Singaporean study only employed 16 participants, which may not be an adequate representation of the community. As a side note, the comparison of gut profiles across studies is inevitably affected by differences in sample and data processing^13,46^.

This study is not free from limitations. Firstly, we did not obtain information on plant-based food intake other than *ulam* consumption. Although we detected the significance of *ulam* on the GM, it has to be noted that *ulam* may not be a proxy for overall plant-based food consumption frequency in all ethnic groups, especially considering the skewed preference for *ulam* across ethnicity in Malaysia^47^. Also, the use of week-long food recall questionnaire instead of a comprehensive food diary to record the participants’ dietary habit prevents an in-depth GM-dietary association from being conducted. However, an in-depth food diary was deemed inappropriate due to concerns over participants’ compliance. Additionally, the absence of a lifestyle food intake analysis presents a possibility that some participants did not practice their typical dietary habit during the week of sampling. Nevertheless, dietary variation across ethnicity in Malaysia has been reported before^48,49^, minimizing the possibility that the significant dietary differences across ethnicity observed in this study was due to irregularity in the dietary patterns of the participants during the sampling week. Furthermore, all the information used in this study, except for age, BP and BMI, were based on self-reported answers, which would have undoubtedly exposed our data through recall bias^50^.

In conclusion, the influence of ethnicity on the gut microbiota was detected from a community living in the same geographical region. This influence could be traced to the collective effect of multiple lifestyle factors exerting subtle yet distinct differences across ethnicity. Ethnicity, therefore, serves as a proxy for lifestyle and dietary variations across different ethnic groups living in the same community. Future studies on the GM should consider the impact of ethnicity to ensure valid interpretation of their study outcome.

## Supplementary Information

Supplementary Information

## Data Availability

The raw sequence data has been uploaded into NCBI sequence read archive (SRA) BioProject no. PRJNA631204.

## References

[1] Tyakht AV, Kostryukova ES, Popenko AS, Belenikin MS, Pavlenko AV (2013). Human gut microbiota community structures in urban and rural populations in Russia. Nat. Commun..

[2] He Y, Wu W, Zheng H-M, Li P, McDonald D (2018). Regional variation limits applications of healthy gut microbiome reference ranges and disease models. Nat. Med..

[3] Davenport ER, Mizrahi-Man O, Michelini K, Barreiro LB, Ober C (2014). Seasonal variation in human gut microbiome composition. PLoS ONE.

[4] Koliada A, Moseiko V, Romanenko M, Piven L, Lushchak O (2020). Seasonal variation in gut microbiota composition: Cross-sectional evidence from Ukrainian population. BMC Microbiol..

[5] Deschasaux M, Bouter KE, Prodan A, Levin E, Groen AK (2018). Depicting the composition of gut microbiota in a population with varied ethnic origins but shared geography. Nat. Med..

[6] Brooks A. W., Priya S., Blekhman R., Bordenstein S. R. Gut microbiota diversity across ethnicities in the United States. *PLoS Biol*. **16**(12) (2018). 10.1371/journal.pbio.2006842 PMC6279019 30513082

[7] Peters BA, Yi SS, Beasley JM, Cobbs EN, Choi HS (2020). US nativity and dietary acculturation impact the gut microbiome in a diverse US population. ISME J..

[8] Abidin M, Habidin NF, Salleh MYY, Hassan P, Yaacob HRM (2016). Assimilation of the Malay culture towards the straights of Chinese community in the state of Kelantan: Study in Kampung Pasir Parit, Chetok, Pasir Mas, Kelantan. Int. J. Acad. Res. Bus Soc. Sci..

[9] Lee R. L. M. Malaysian identities and mélange food cultures. *J. Intercult. Stud*. **38**(2), 139–154. 10.1080/07256868.2017.1289907 (2017).

[10] Chong CW, Ahmad AF, Lim YAL, Teh CSJ, Yap IKS (2015). Effect of ethnicity and socioeconomic variation to the gut microbiota composition among pre-adolescent in Malaysia. Sci. Rep..

[11] Khine WWT, Zhang Y, Goie GJY, Wong MS, Liong M (2019). Gut microbiome of pre-adolescent children of two ethnicities residing in three distant cities. Sci. Rep..

[12] Partap U, Young EH, Allotey P, Soyiri IN, Jahan N (2017). HDSS profile: The South East Asia community observatory health and demographic surveillance system (SEACO HDSS). Int. J. Epidemiol..

[13] Costea PI, Zeller G, Sunagawa S, Pelletier E, Alberti A (2017). Towards standards for human fecal sample processing in metagenomic studies. Nat. Biotechnol..

[14] Callahan BJ, McMurdie PJ, Rosen MJ, Han AW, Johnson AJA (2016). DADA2: High-resolution sample inference from Illumina amplicon data. Nat. Methods..

[15] Bowman J. S., Ducklow H. W. Microbial communities can be described by metabolic structure: a general framework and application to a seasonally variable, depth-stratified microbial community from the coastal West Antarctic Peninsula. *PLoS One*. **10**(8) (2015). 10.1371/journal.pone.0135868 PMC4540456 26285202

[16] Karp PD, Riley M, Saier M, Paulsen IT, Paley SM (2000). The EcoCyc and MetaCyc databases. Nucleic Acids Res..

[17] McMurdie PJ, Holmes S (2013). phyloseq: an R package for reproducible interactive analysis and graphics of microbiome census data. PLoS ONE.

[18] Fernandes AD, Macklaim JM, Linn TG, Reid G, Gloor GB (2013). ANOVA-like differential expression (ALDEx) analysis for mixed population RNA-Seq. PLoS ONE.

[19] Gloor GB, Macklaim JM, Pawlowsky-Glahn V, Egozcue JJ (2017). Microbiome datasets are compositional: And this is not optional. Front. Microbiol..

[20] Dixon P (2003). VEGAN, a package of R functions for community ecology. J. Veg. Sci..

[21] Bates D., Mächler M., Bolker B. M., Walker S. C. Fitting linear mixed-effects models using lme4. *J. Stat, Softw*. **67**(1), 1 (2015).

[22] Holmes I, Harris K, Quince C (2012). Dirichlet multinomial mixtures: generative models for microbial metagenomics. PLoS ONE.

[23] Arumugam M, Raes J, Pelletier E, Le Paslier D, Yamada T (2011). Enterotypes of the human gut microbiome. Nature.

[24] David LA, Maurice CF, Carmody RN, Gootenberg DB, Button JE (2014). Diet rapidly and reproducibly alters the human gut microbiome. Nature.

[25] Chen J, Ryu E, Hathcock M, Ballman K, Chia N (2016). Impact of demographics on human gut microbial diversity in a US Midwest population. PeerJ..

[26] Reihani S, Azhar M (2012). Antioxidant activity and total phenolic content in aqueous extracts of selected traditional Malay salads (Ulam). Int. Food Res. J..

[27] Bachok MF, Yusof BN, Ismail A, Hamid AA (2014). Effectiveness of traditional Malaysian vegetables (ulam) in modulating blood glucose levels. Asia Pac. J. Clin. Nutr..

[28] Larsen N, Vogensen FK, van den Berg FWJ, Nielsen DS, Andreasen AS (2010). Gut microbiota in human adults with type 2 diabetes differs from non-diabetic adults. PLoS ONE.

[29] Hussein, Z., Taher, S. W., Gilcharan Singh H. K., Chee Siew Swee, W. Diabetes care in Malaysia: problems, new models, and solutions. *Ann. Glob. Health*. **81**(6), 851–862. Doi:10.1016/j.aogh.2015.12.016 (2015). 10.1016/j.aogh.2015.12.016 27108152

[30] Linares DM, Gómez C, Renes E, Fresno JM, Tornadijo ME (2017). Lactic acid bacteria and bifidobacteria with potential to design natural biofunctional health-promoting dairy foods. Front. Microbiol..

[31] Stearns JC, Zulyniak MA, de Souza RJ, Campbell NC, Fontes M (2017). Ethnic and diet-related differences in the healthy infant microbiome. Genome Med..

[32] Tang M, Frank DN, Tshefu A, Lokangaka A, Goudar SS (1848). Different gut microbial profiles in Sub-Saharan African and South Asian women of childbearing age are primarily associated with dietary intakes. Front. Microbiol..

[33] Gurung M, Li Z, You H, Rodrigues R, Jump DB (2020). Role of gut microbiota in type 2 diabetes pathophysiology. EBioMedicine..

[34] Rothschild D, Weissbrod O, Barkan E, Kurilshikov A, Korem T (2018). Environment dominates over host genetics in shaping human gut microbiota. Nature.

[35] Huttenhower C, Gevers D, Knight R, Abubucker S, Badger JH (2012). Structure, function and diversity of the healthy human microbiome. Nature.

[36] Rodriguez Cetina Biefer H., Vasudevan A., Elkhal A. Aspects of tryptophan and nicotinamide adenine dinucleotide in immunity: A new twist in an old tale. *Int. J. Tryptophan. Res*. **10**, 1. Doi:10.1177/1178646917713491 (2017). 10.1177/1178646917713491 PMC5476425 28659716

[37] Friedman M (2018). Analysis, nutrition, and health benefits of tryptophan. Int. J. Tryptophan. Res..

[38] Ohlendorf DH, Orville AM, Lipscomb JD (1994). Structure of protocatechuate 3,4-dioxygenase from *Pseudomonas aeruginosa* at 2.15 Å resolution. J. Mol. Biol..

[39] Phillips DH (1999). Polycyclic aromatic hydrocarbons in the diet. Mutat. Res. Genet. Toxicol. Environ. Mutagen..

[40] Yatsunenko T, Rey FE, Manary MJ, Trehan I, Dominguez-Bello MG (2012). Human gut microbiome viewed across age and geography. Nature.

[41] Forslund K, Hildebrand F, Nielsen T, Falony G, Le Chatelier E (2015). Disentangling type 2 diabetes and metformin treatment signatures in the human gut microbiota. Nature.

[42] Li J, Zhao F, Wang Y, Chen J, Tao J (2017). Gut microbiota dysbiosis contributes to the development of hypertension. Microbiome..

[43] Ridaura VK, Faith JJ, Rey FE, Cheng J, Duncan AE (2013). Gut microbiota from twins discordant for obesity modulate metabolism in mice. Science.

[44] Gevers D, Knight R, Petrosino JF, Huang K, McGuire AL (2012). The Human Microbiome Project: A community resource for the healthy human microbiome. PLoS Biol..

[45] Jain A, Li XH, Chen WN (2018). Similarities and differences in gut microbiome composition correlate with dietary patterns of Indian and Chinese adults. AMB Express..

[46] Pollock J, Glendinning L, Wisedchanwet T, Watson M (2018). The madness of microbiome: Attempting to find consensus “best practice” for 16S microbiome studies. Appl. Environ. Microbiol..

[47] Izzah AN, Aminah A, Pauzi AM, Lee Y, Rozita WW (2012). Patterns of fruits and vegetable consumption among adults of different ethnics in Selangor Malaysia. Int. Food Res. J..

[48] Abdullah NF, Teo PS, Foo LH (2016). Ethnic differences in the food intake patterns and its associated factors of adolescents in Kelantan, Malaysia. Nutrients..

[49] Chee-Beng T (2000). Ethnic identities and national identities: Some examples from Malaysia. Identities..

[50] Althubaiti A (2016). Information bias in health research: Definition, pitfalls, and adjustment methods. J. Multidiscip. Healthc..

