# Supplementary material for: Ethnicity influences the gut microbiota of individuals sharing a geographical location: a cross-sectional study from a middle-income country
Source: Sci Rep. 2021 Jan 29;11:2618. doi: 10.1038/s41598-021-82311-3 (PMC7846579; doi:10.1038/s41598-021-82311-3)
Supplement: Supplementary file 1 — Supplementary Information [file 41598_2021_82311_MOESM1_ESM.pdf]

Supplementary Information for:

Title: Ethnicity influences the gut microbiota of individuals sharing a geographical location: A cross-sectional study from a middle-income country

Running title: gut microbiota of a Malaysian community

Dwiyanto, J[1*], Hussain, MH[1], Reidpath, D[2, 3], Ong, KS[1], Qasim, A[1, 4], Lee, SWH[5], Lee, SM[1], Foo, SC[1], Chong, CW[5] & Rahman, S[1, 6*]

[1] School of Science, Monash University Malaysia, Bandar Sunway, Malaysia

[2] Health System and Population Studies Division, International Centre for Diarrhoeal Disease Research, Bangladesh, Dhaka, Bangladesh

[3] South East Asia Community Observatory, Segamat, Malaysia

[4] Genomics Facility, Monash University Malaysia, Bandar Sunway, Malaysia

[5] School of Pharmacy, Monash University Malaysia, Bandar Sunway, Malaysia

[6] Tropical Medicine and Biology Platform, Monash University Malaysia, Bandar Sunway, Malaysia

Address for correspondence:

Jacky Dwiyanto, email: jacky.dwiyanto@monash.edu

Sadequr Rahman, email: sadequr.rahman@monash.edu

School of Science, Monash University Malaysia, Jalan Lagoon Selatan, 47500 Bandar Sunway, Selangor Darul Ehsan, Malaysia

Supplementary Table S1. Age distribution of the study participant across ethnicity

Ethnicity	10-28	29-48	49-59	60-83	Chi-square P-value
Chinese	12	7	23	23	0.01
Indian	7	18	12	12	0.17
Jakun	22	20	6	6	0.00
Malay	13	10	10	13	0.85

Supplementary Table S2. Lifestyle factors obtained from Segamat community participants

Category	Factors	PERMANOVA		Level	Chinese		Jakun		Indian		Malay		N	Chi Sq
		Effect size	P		n	%	n	%	n	%	n	%		P-value
Dietary preference	Ulam	0.01	0.01	No	42	0.47	10	0.11	26	0.29	11	0.12	89	0.00
				Yes	23	0.18	44	0.35	23	0.18	35	0.28	125	0.02
	Pork	0.01	0.01	No	4	0.03	37	0.28	45	0.34	46	0.35	132	0.00
				Yes	61	0.74	17	0.21	4	0.05	0	0.00	82	0.00
	Beef	0.01	0.01	No	57	0.42	27	0.20	42	0.31	11	0.08	137	0.00
				Yes	8	0.10	27	0.35	7	0.09	35	0.45	77	0.00
	Chicken	0.00	0.40	No	3	0.16	6	0.32	6	0.32	4	0.21	19	0.70
				Yes	62	0.32	48	0.25	43	0.22	42	0.22	195	0.16
	Fish	0.01	0.04	No	6	0.22	16	0.59	4	0.15	1	0.04	27	0.00
				Yes	59	0.32	38	0.20	45	0.24	45	0.24	187	0.17
	Raw food	0.01	0.06	No	64	0.32	49	0.25	39	0.19	46	0.23	198	0.08
				Yes	1	0.06	5	0.31	10	0.63	0	0.00	16	0.00
	Probiotics	0.01	0.21	No	42	0.27	36	0.23	33	0.21	43	0.28	154	0.62
				Yes	21	0.36	18	0.31	16	0.28	3	0.05	58	0.00
	Fermented food	0.01	0.15	No	5	0.14	10	0.29	16	0.46	4	0.11	35	0.02
				Yes	56	0.32	42	0.24	33	0.32	42	0.24	173	0.09
	Fruits	0.01	0.13	No	6	0.26	13	0.57	0	0.00	4	0.17	23	0.00
				Yes	57	0.31	41	0.23	45	0.25	39	0.21	182	0.23
	Coffee	0.01	0.20	No	29	0.26	31	0.28	22	0.20	27	0.25	109	0.65
				Yes	32	0.32	23	0.23	27	0.27	19	0.19	101	0.29
	Tea	0.01	0.09	No	29	0.30	20	0.21	27	0.28	20	0.21	96	0.43
				Yes	32	0.28	34	0.29	22	0.19	26	0.23	114	0.36
Hygiene	Toilet type	0.04	0.00	Flush – sewerage	34	0.44	5	0.06	25	0.32	14	0.18	78	0.00
				Flush - septic tank	31	0.31	17	0.17	22	0.22	30	0.30	100	0.15
				Pourflush	0	0.00	6	1.00	0	0.00	0	0.00	6	0.00
				Borehole	0	0.00	19	0.83	2	0.09	2	0.09	23	0.00
				Bucket latrine	0	0.00	4	1.00	0	0.00	0	0.00	4	0.01
				Others	0	0.00	3	1.00	0	0.00	0	0.00	3	0.03
	Toilet nature	0.02	0.00	Squat	7	0.09	50	0.61	19	0.23	6	0.07	82	0.00
				Sit	25	0.43	0	0.00	21	0.36	12	0.21	58	0.00
				Both	33	0.45	4	0.05	9	0.12	28	0.38	74	0.00
	Toilet location	0.01	0.46	Inside	61	0.34	38	0.21	47	0.26	32	0.18	178	0.01
				Outside	2	0.08	13	0.50	2	0.08	9	0.35	26	0.00
				Both	2	0.20	3	0.30	0	0.00	5	0.50	10	0.16
	Bathing water source	0.02	0.00	Piped	65	0.34	30	0.16	49	0.26	46	0.24	190	0.00
				Pond/Stream	0	0.00	16	1.00	0	0.00	0	0.00	16	0.00
				Others	0	0.00	8	1.00	0	0.00	0	0.00	8	0.00
	Drinking water source	0.03	0.00	Indoor piped	65	0.35	28	0.15	48	0.26	46	0.25	187	0.00
				Piped in yard	0	0.00	0	0.00	1	1.00	0	0.00	1	0.39
				Bottled	0	0.00	2	1.00	0	0.00	0	0.00	2	0.11
				Pond/Stream	0	0.00	15	1.00	0	0.00	0	0.00	15	0.00
				Others	0	0.00	9	1.00	0	0.00	0	0.00	9	0.00

	Handwashing	0.03	0.00	Never	21	0.95	1	0.05	0	0.00	0	0.00	22	0.00
				Sometimes	11	0.85	1	0.08	1	0.08	0	0.00	13	0.00
				Usually	12	0.27	12	0.27	9	0.20	12	0.27	45	0.90
				Half of the time	5	0.83	1	0.17	0	0.00	0	0.00	6	0.01
				Always	16	0.13	39	0.30	39	0.30	34	0.27	128	0.01
	Utensil preference	0.03	0.00	Barehand	1	0.01	40	0.36	36	0.32	34	0.31	111	0.00
				Yes	61	0.88	7	0.10	1	0.01	0	0.00	69	0.00
				Both	3	0.09	6	0.18	12	0.36	12	0.36	33	0.06
				Blank data	0	0.00	1	1.00	0	0.00	0	0.00	1	0.39
Health condition	Allergy	0.00	0.43	No	59	0.35	40	0.24	36	0.21	34	0.20	169	0.03
				Yes	6	0.13	14	0.31	13	0.29	12	0.27	45	0.33
	Cigarette	0.00	0.41	No	58	0.33	40	0.23	39	0.22	37	0.21	174	0.09
				Yes	7	0.18	14	0.35	10	0.25	9	0.23	40	0.46
	Blood pressure	0.02	0.43	Normal (<120/80)	26	0.35	15	0.20	18	0.24	15	0.20	74	0.22
				Elevated (120 –129/<80)	14	0.36	9	0.23	9	0.23	7	0.18	39	0.43
				Stage 1 (130 – 139/80 – 89)	10	0.22	15	0.33	10	0.22	10	0.22	45	0.64
				Stage 2 (≥140/90)	15	0.28	13	0.25	12	0.23	13	0.25	53	0.95
				Crisis (≥180/120)	0	0.00	2	0.67	0	0.00	1	0.33	3	0.30
				Hypertensive (>129/>79)	25	0.25	30	0.30	22	0.22	24	0.24	101	0.71
	Body mass index	0.02	0.08	Underweight (<18.5)	6	0.27	8	0.36	2	0.09	6	0.27	22	0.33
				Healthy (18.5 – 24.99)	38	0.40	23	0.24	13	0.14	20	0.21	94	0.00
				Overweight (25 – 29.99)	15	0.27	10	0.18	19	0.35	11	0.20	55	0.30
				Obese (>30)	6	0.14	13	0.30	15	0.35	9	0.21	43	0.21
	Medication	0.01	0.00	No	38	0.27	42	0.30	26	0.18	35	0.25	141	0.27
				Yes	27	0.37	12	0.16	23	0.32	11	0.15	73	0.02
	Any chronic disease	0.01	0.02	No	39	0.28	38	0.27	27	0.19	35	0.25	139	0.47
				Yes	26	0.35	16	0.21	22	0.29	11	0.15	75	0.07
	Diabetes	0.01	0.01	No	59	0.31	52	0.28	34	0.18	43	0.23	188	0.06
				Yes	6	0.23	2	0.08	15	0.58	3	0.12	26	0.00
	Cholesterol	0.00	0.37	No	52	0.28	52	0.28	40	0.21	43	0.23	187	0.48
				Yes	13	0.48	2	0.07	9	0.33	3	0.11	27	0.01
	BSS Scale	0.01	0.00	1	1	0.11	6	0.67	1	0.11	1	0.11	9	0.04
				2	10	0.31	4	0.13	9	0.28	9	0.28	32	0.43
				3	11	0.17	22	0.35	20	0.32	10	0.16	63	0.07
				4	21	0.43	9	0.18	7	0.14	12	0.24	49	0.02
				5	10	0.40	6	0.24	2	0.08	7	0.28	25	0.16
				6	7	0.29	6	0.25	8	0.33	3	0.13	24	0.51
				7	5	0.42	1	0.08	2	0.17	4	0.33	12	0.34
	Surgical history	0.01	0.15	No	65	0.32	52	0.25	45	0.22	44	0.21	206	0.14
				Yes	3	0.27	2	0.18	4	0.36	2	0.18	11	0.80
Family history of chronic disease	Any chronic disease	0.00	0.36	No	24	0.35	15	0.22	16	0.23	14	0.20	69	0.30
				Yes	41	0.28	39	0.27	33	0.23	32	0.22	145	0.65
	Diabetes	0.00	0.42	No	43	0.31	41	0.30	24	0.18	29	0.21	137	0.06

		0.00	0.42	Yes	22	0.29	13	0.17	25	0.32	17	0.22	77	0.22
	Hypertension	0.00	0.65	No	34	0.31	23	0.21	29	0.27	22	0.20	108	0.32
				Yes	31	0.29	31	0.29	20	0.19	24	0.23	106	0.34
	Cholesterol	0.00	0.46	No	55	0.29	48	0.26	44	0.23	41	0.22	188	0.50
				Yes	10	0.38	6	0.23	5	0.19	5	0.19	26	0.45
	Other chronic conditions	0.00	0.91	No	55	0.29	43	0.23	46	0.24	44	0.23	188	0.59
				Yes	10	0.38	11	0.42	3	0.12	2	0.08	26	0.02
Other	Proximity to animals	0.01	0.23	No	35	0.38	17	0.18	19	0.20	22	0.24	93	0.04
				Yes	30	0.25	37	0.31	30	0.25	24	0.20	121	0.42
	Number of people in household	0.02	0.07	1 – 3	18	0.29	13	0.21	20	0.32	11	0.18	62	0.33
				4 – 5	20	0.24	22	0.27	17	0.20	24	0.29	83	0.73
				6	10	0.29	11	0.32	5	0.15	8	0.24	34	0.48
				7 – 16	17	0.49	8	0.23	7	0.20	3	0.09	35	0.01
	Healthy group	0.10	0.00	No	51	0.31	41	0.25	39	0.23	36	0.22	167	0.39
				Yes	14	0.30	13	0.28	10	0.21	10	0.21	47	0.78

Blood pressure was categorized using categories based on the American Heart Association (https://www.heart.org/en/health-topics/high-blood-pressure/understanding-blood-pressure-readings)

Body mass index was categorized using categories based on Malaysia Ministry of Health classification (http://www.myhealth.gov.my/en/bmi/)

Supplementary Table S3. Criteria used for selecting healthy participants

Factor	Value
Age	>10
BMI	16-30 (Underweight – Overweight)
BP	Lower than 140/90 (Stage 1)
Blood sugar	Not measured
Drug	No current drug intake
Surgery	No surgical history in the past one year
Bristol Stool Scale	Between 3 and 5
Disease	No reported chronic condition

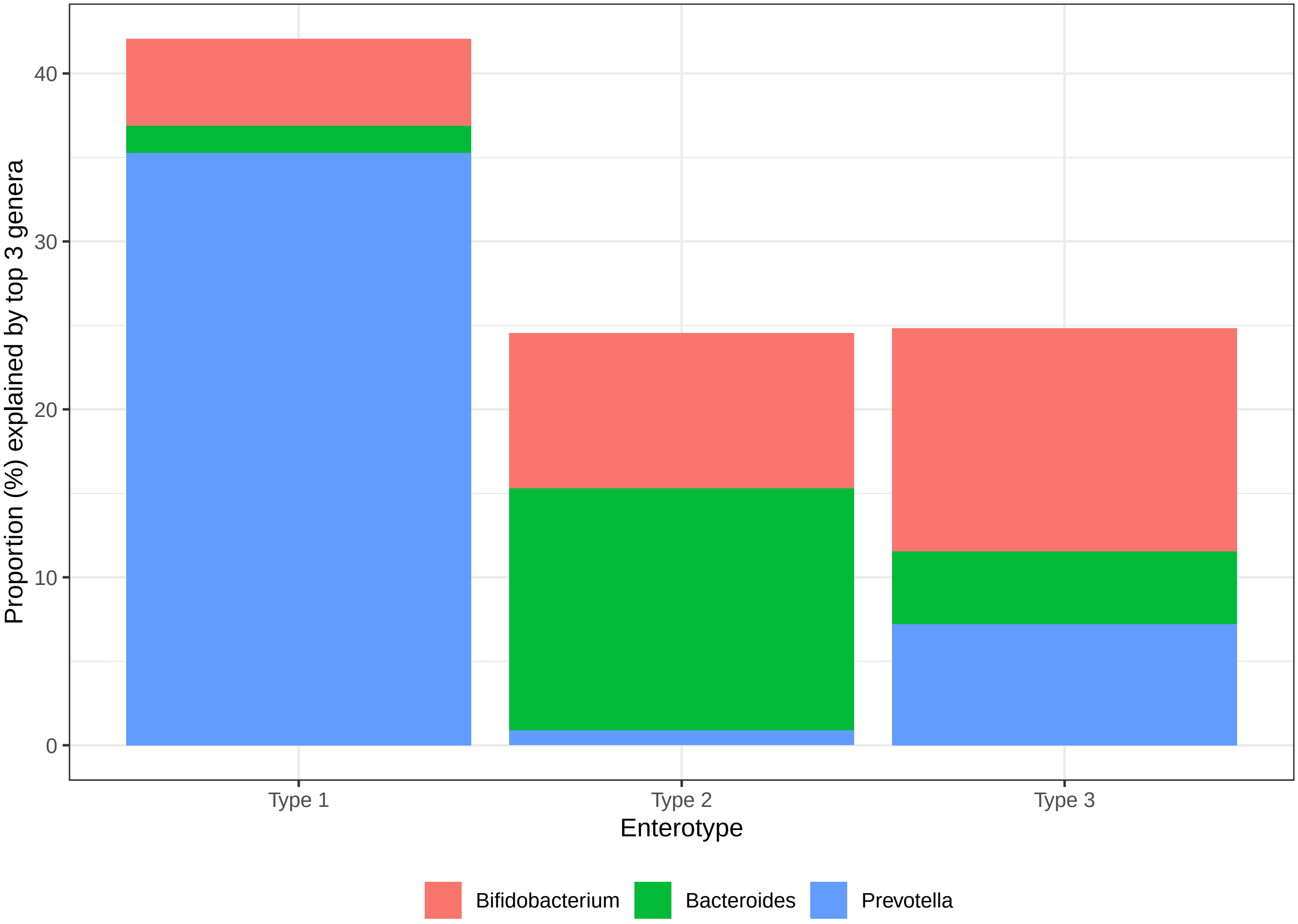

Supplementary Figure S1. Proportion of the top 3 genera of each enterotype detected based on Dirichlet Multinomial Analysis
